# Post-operative Achilles Tendinopathy Following Surgical Repair of Achilles Tendon Rupture Treated With Platelet-Rich Plasma: A Report of Two Cases

**DOI:** 10.7759/cureus.95250

**Published:** 2025-10-23

**Authors:** Jake Van Weezep, Daniella Rivera, Alexander Kim

**Affiliations:** 1 Family Medicine, David Grant Medical Center, Travis Air Force Base, Fairfield, USA; 2 Sports Medicine, David Grant Medical Center, Travis Air Force Base, Fairfield, USA

**Keywords:** achilles repair, achilles rupture, achilles tendinopathy, achilles tendon injury, platelet-rich plasma/prp, post-op pain management

## Abstract

Post-operative Achilles tendinopathy (AT) can cause persistent, debilitating posterior ankle pain. The optimal treatment for recalcitrant AT following surgical repair of Achilles tendon rupture has not been well-established. Conservative management includes early weight-bearing and mobilization with structured physical therapy (PT) and a progressive return to activity. Despite completion of conservative measures, symptoms may persist long after surgical repair. Platelet-rich plasma (PRP) has been shown to effectively treat painful MSK conditions, including AT. There have been no published studies assessing the efficacy or safety of PRP injections to treat post-operative AT after repair of tendon rupture. We present two cases of refractory AT after surgical repair of a ruptured Achilles tendon treated with ultrasound (US) guided PRP injections.

## Introduction

Achilles tendinopathy (AT), a common injury to the Achilles tendon, may cause posterior ankle and heel pain that can be challenging to treat. AT often occurs due to chronic overuse but may occur after surgical repair of an Achilles tendon rupture (ATR) [1]. Optimal treatments for AT following surgical repair for ATR are not well established. We report the first published cases of refractory AT after surgical repair of ATR successfully treated with platelet-rich plasma (PRP).

## Case presentation

A 37- and 38-year-old man presented separately with 10 and nine years of persistent right-sided post-operative posterior ankle pain. Both had sustained an acute, sports-related, right-sided ATR several years prior to presentation, and underwent an immediate post-injury open surgical repair. They underwent extensive supervised physical therapy (PT) after surgery with moderate improvement in pain, but without resolution. On presentation to the clinic, both patients reported more than five years of worsening Achilles pain localized to the site of injury and surgical repair. Both were active-duty military service members with no history of metabolic disturbance, and reported that their pain was exacerbated by running, a requirement to maintain service fitness standards. Due to progressive pain, both men were unable to continue their fitness training, prompting them to seek additional treatment.

**Figure 1:**
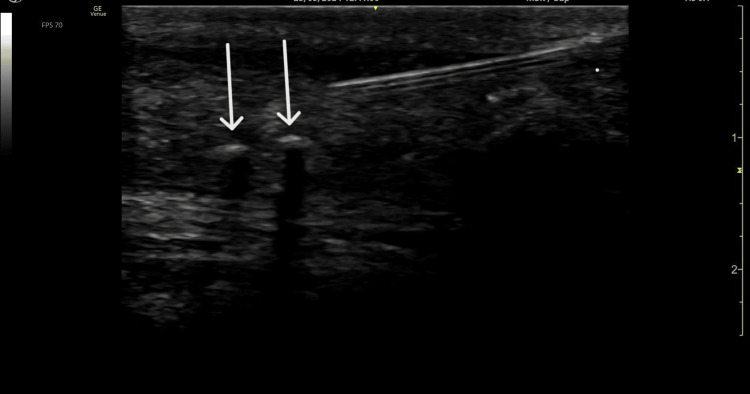
Ultrasound of the Achilles tendon of Patient 1 in long-axis view showing marked tendon thickening with anechoic voids and intra-tendinous calcifications consistent with tendinopathy and post-operative change.

Physical examination of both men revealed tenderness to palpation at the midsubstance of the right Achilles tendon over the site of repair, with pain exacerbated by resisted ankle plantar flexion. Thompson’s squeeze test and Achilles reflex were normal. Musculoskeletal ultrasound (US) demonstrated a thickened midsubstance Achilles tendon with multiple anechoic voids and calcifications, suggesting midsubstance tendinopathy (Figure 1), though these findings can be typical in post-operative ATR [2]. Both patients were prescribed supervised PT and a home exercise program focusing on progressive eccentric loading. Both returned after several months without any improvement, and both reported debilitating pain as an eight out of 10 on the visual analog scale (VAS).

**Figure 2:**
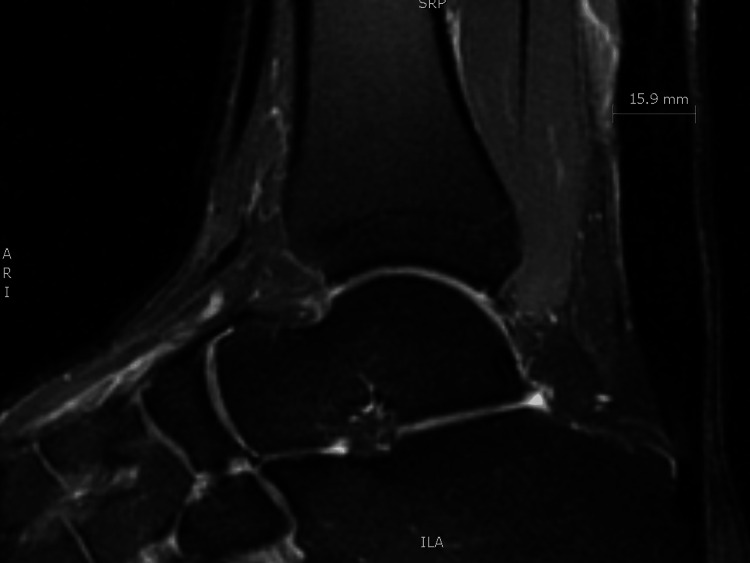
Ankle MRI of Patient 1 with T2 fat suppression (PD FS) sagittal image showing thickening of the Achilles tendon.

Given the prior history of ATR with surgical repair and a paucity of data regarding PRP in the post-operative setting, repeat magnetic resonance imaging (MRI) was obtained to definitively rule out partial tearing prior to injection therapy. These studies revealed intact Achilles tendons with moderate thickening of the midsubstance with no evidence of tearing (Figure 2). As their pain was chronically debilitating, various treatment options were discussed. Both men preferred to avoid additional surgical intervention. Corticosteroid injection was considered but deferred, given the history of tendon rupture and evidence of tendinopathy on imaging. The shared decision was made for intratendinous PRP injection. Thirty mL of whole blood was processed with an EmCyte PurePRP system (EmCyte Corporation, Fort Myers, FL) to produce 4 mL of leukocyte-poor PRP (LP-PRP). LP-PRP is preferred in our clinic as it has demonstrated similar efficacy with less post-procedural pain compared to leukocyte-rich PRP (LR-PRP) in treating AT [3]. After sterilizing the skin, 3 mL of PRP was injected into the right Achilles tendon of both patients under US guidance (GE Venue Go, L4-12L-RS linear array transducer, GE HealthCare Technologies Inc., Chicago, IL) with a 22-gauge 1.5-inch needle. Topical anesthesia was not used before or during the injection. Both patients tolerated the procedure without complications. They were instructed to return to light duty at day 7, begin light rehabilitation exercise at 14 days using the previously prescribed home exercise program, and to avoid non-steroidal anti-inflammatory drugs (NSAIDs) for 14 days.

Both patients reported significant improvement at follow-up visits. One reported a 50% reduction in pain at 12-week follow-up, enabling a return to running. His 50% pain reduction was sustained at 24 weeks, and he was able to advance his fitness routine. The other patient reported 75% improvement of his pain at 12-week follow-up, which was sustained at 24 weeks, enabling a return to fitness.

## Discussion

AT is an injury affecting the midsubstance or distal insertion of the Achilles tendon, causing pain and functional limitations [4,5]. Repetitive high-energy loads cause tendon elongation and fatigue failure, predisposing an athlete to ATR [6]. ATR may occur due to acute loading during acceleration or deceleration or a single, high-load eccentric event. ATR can be managed operatively or non-operatively based on several factors and patient goals [6]. Operative management typically results in faster return-to-play and lower rates of re-rupture, which may be preferred in younger, active patients [6].

Despite the benefits of surgical repair of ATR, some may experience persistent symptoms following surgery. One prospective study showed that 12 months after surgical repair of ATR, 22% of patients reported persistent pain and 16% reported issues with mobility [1]. Roughly 20% of athletes are unable to return to sport following surgical repair, and many patients require years to return to pre-injury baseline levels of performance [6]. Though the mechanism is not completely understood, post-operative AT after repair of ATR may occur secondary to post-repair neovascularization, fibrous degeneration, and granuloma formation [7].

PRP is emerging as an efficacious treatment option for Achilles injuries [5,8,9]. It has been shown in multiple studies to be an effective treatment option for chronic Achilles tendinopathy not associated with rupture or surgery [4,5,8]. Evidence supporting use in Achilles tendon rupture is emerging as well. One systematic review and meta-analysis of ATR cases managed non-operatively with PRP injections reported significant improvement in ankle range of motion, strength, and calf circumference [9]. A recent case reported a patient with ATR who returned to sport three months post-injury after treatment with combined freeze-dried platelet-derived factor concentrate and early rehabilitation following surgical repair. This is a significantly shorter course than typically expected, demonstrating the potential benefit of platelet products used post-operatively [10].

## Conclusions

To the best of the authors’ knowledge, these are the first reported cases of prolonged post-operative AT after surgical repair of ATR successfully treated with PRP. In these cases, PRP injection was shown to be safe and significantly improved both patients’ pain and function, obviating the need for further surgical consultation. Both patients had years of persistent pain after surgery despite conservative management, highlighting the efficacy of PRP in refractory cases. Though the exact mechanism of PRP in treating post-operative tendinopathy is unclear, PRP may promote histopathological recovery of the Achilles tendon by shortening the inflammatory phase and accelerating tendon healing. Given the structural changes that occur after ATR repair, PRP may improve the structural integrity and strength of the tendon, resulting in improved pain and function. While the results presented here are limited in power by the small number of patients involved, the improvement in pain and function suggests further studies are warranted to more definitively establish the efficacy of treatment and determine the mechanism of action, optimal timing, and PRP formulation to maximize treatment benefit.
